# Carbon Offsets: Growing Pains in a Growing Market

**DOI:** 10.1289/ehp.117-a62

**Published:** 2009-02

**Authors:** Charles W. Schmidt

There’s a market growing in the United States, but unlike markets that trade in tangible commodities, this one trades in the absence of something no one wants: greenhouse gases in the atmosphere. Hundreds of companies make it possible for individuals, organizations, businesses, and even events such as rock music festivals to proclaim themselves carbon-neutral by paying someone else to reduce their emissions. Worried about your carbon footprint? No problem. For fees of US$2–50 per ton of “avoided emissions,” an offset provider will funnel your money into an activity or technology that keeps greenhouse gases out of the atmosphere. The question is, are offset buyers really getting what they paid for?

## The Offset Market

According to the nonprofit Ecosystems Market place, which tracks the green services industry, the global market for voluntary offsets—which don’t count toward compliance with mandated emissions reductions such as those required by the Kyoto Protocol—more than tripled from 2006 to 2007, reaching US$331 million. This nonregulated “over-the-counter” market is accessible to any person, business, or group wanting to minimize its carbon footprint; businesses accounted for roughly 80% of the trade. The over-the-counter market is dwarfed by that within a vast compliance framework for cutting emissions—the Clean Development Mechanism (CDM)—through which companies bound to the Kyoto Protocol can purchase offsets from projects in the developing world. Unlike over-the-counter purchases, CDM offsets (known as “certified emission reductions,” or CERs) count toward compliance with the Kyoto Protocol’s legally binding emission reduction targets. According to figures published 14 January 2009 by the Oslo-based market analysis firm Point Carbon, the CDM did US$32 billion in CER trade in 2008, more than double the previous year’s figure.

Offset sales in the United States are poised to accelerate, assuming a compliance framework for regulating greenhouse gases emerges to meet President Obama’s climate goals. During his address to the bipartisan Governors’ Global Climate Summit on 18 November 2008, then President-Elect Obama proposed an 80% reduction in global warming emissions by 2050. Offsets already play a part in smaller compliance programs in the United States. Among them is the Regional Greenhouse Gas Initiative (RGGI), a cooperative effort among 10 Northeastern states that allows utilities to apply offsets toward their compliance target of a 10% cut in emissions between 2009 and 2018.

Offset projects can take many forms. They might be landfills that capture methane; they might be tree farms in the tropics; or they might be renewable energy installations such as wind farms. In order for them to be considered viable, offset projects must satisfy four key requirements: They have to be “additional,” meaning they come from activities that wouldn’t happen in the absence of an offsets incentive; they have to be quantifiable, meaning they measurably reduce emissions; they have to be permanent, meaning the greenhouse gases they keep out of the atmosphere won’t be released later; and they have to be real, meaning they can be verified by third-party inspectors.

Yet, mounting evidence shows many projects don’t meet these requirements, especially that of additionality. On 20 October 2008, *Wall Street Journal* reporter Jeffrey Ball reported that landfill operators across the country were selling offsets for methane capture projects that had been ongoing for years. The landfills in question were selling methane for fuel, a lucrative endeavor that also happens to be climate-friendly, as the carbon dioxide (CO_2_) released by methane combustion is far less dangerous to the climate than methane itself. The Cape May County Municipal Utilities Authority in New Jersey, which raised $427,475 from offsets sold in the first nine months of 2008 alone, had been selling methane for more than a decade. In terms of additionality, those who bought these offsets contributed nothing to further emissions reductions since the utility’s methane capture system was implemented far in advance of any likely intention to sell offsets.

David Victor, who directs the program on energy and sustainable development at Stanford University’s Freeman Spogli Institute for International Studies, claims CDM offsets are “mostly bogus.” In “A Realistic Policy on International Carbon Offsets,” a working paper issued in April 2008, Victor and coauthor Michael Wara, a research fellow and lecturer at Stanford Law School, described their investigation into industrial energy projects in China—such as new hydro, wind, and natural gas plants—which account for the vast majority of CER credits issued today. According to their investigation, most of these projects have applied for approval under the CDM, which would make them eligible for payments from Kyoto signatories, even though China’s latest five-year economic plan calls for major investments in alternative energy to shift the country away from coal.

“Taken collectively . . . these individual applications for credit amount to a claim that the hydro, wind, and natural gas elements of the power sector in China would not be growing at all without help from CDM,” the authors wrote. “This broader implication is simply implausible in light of [China’s] state policies.” The specific concern, the Stanford researchers noted, is that China’s energy projects don’t meet requirements for “regulatory additionality.” In other words, the projects are already required by regulation and would thus proceed even without offset funding. Says Victor, “What we see here are energy developers [in China] who have become quite clever when it comes to filling out the right forms to get CDM credit.”

A similar conclusion was reached by the Government Accountability Office (GAO) in the November 2008 report *International Climate Change Programs: Lessons Learned from the European Union’s Emissions Trading Scheme and the Kyoto Protocol’s Clean Development Mechanism*, which stated that offsets under the Kyoto Protocol had yielded “uncertain” effects on greenhouse emissions, with “limited” contributions to sustainable technology development. The report further stated that “some offset credits were awarded for projects that would have occurred even in the absence of the CDM, despite a rigorous screening process.”

## Regulation Versus Market Certification

Findings such as these have put policy stakeholders on alert. The Federal Trade Commission (FTC), which investigates cases of market deception, warns that offsets carry a high risk of fraud, according to Jim Kohm, associate director of enforcement in the commission’s Bureau of Consumer Protection. Kohm says buyers should be wary of double-counting, in which developers sell multiple offsets for a single project. “We sue people for that,” he says.

The challenge, Kohm adds, is that people buy offsets on faith. “Offsets are not like products that you can touch or feel,” he says. “I might sell you an offset for planting a tree, but how do you know that I haven’t also sold that offset to someone else?” But Kohm also says the FTC’s concerns don’t extend beyond double-counting to additionality unless sellers make false claims that their projects are additional when they aren’t.

As for entities such as the landfill utilities described in the *Wall Street Journal* article, Kohm claims they could only be accused of fraud if they had intentionally misled consumers. “Under the FTC Act you can sell an offset that isn’t additional without breaking the law as long as you are clear about what you are selling,” he explains. “You could say, ‘I’m selling you an offset, but it’s not additional.’” He says the FTC has yet to prosecute a single case of offset fraud but wouldn’t comment as to whether any such cases are in the FTC’s investigative pipeline.

Part of the problem, asserted the GAO in an August 2008 report, *The U.S. Voluntary Market Is Growing, but Quality Assurance Poses Challenges for Market Participants*, is that although federal agencies supply some consumer protection and technical assistance in this area, “no single regulatory body [in the United States] has oversight responsibilities [for the over-the-counter offset market].”

In place of a centralized regulatory framework for offsets, a number of independent, market-based standards have emerged to impose a measure of honesty and integrity on the business. The Voluntary Carbon Standard (VCS), for instance, was created in 2005 by The Climate Group, the International Emissions Trading Association, and the World Economic Forum to ensure that offsets in the voluntary market are additional and permanent. To qualify for VCS approval, projects must pass inspection by third-party verifiers. Approved projects can issue tradable offset credits known as Voluntary Carbon Units.

Those who champion offsets claim consumers can avoid nonviable purchases if they buy from sellers that work through market-based certification programs. David Antinioli, chief executive officer of the Voluntary Carbon Standard Association, which manages the VCS program, stresses that society can and should have faith in market instruments. For example, the Center for Resource Solutions (CRS), a San Francisco–based nonprofit organization that certifies products sold by companies in the green energy sector, bills itself as an organization that helps offset buyers know exactly what they’re purchasing. Certification standards by independent, outside organizations such as the VCS, Gold Standard, and the CRS Green-e Climate program seek to ensure that projects are real, additional, quantifiable, and permanent, and help buyers select offsets that aren’t being double-sold or offered by companies making misleading claims, says Jeff Swenerton, communications director at the CRS.

Bill Burtis, manager of communications and special projects for the Portsmouth, New Hampshire–based environmental group Clean Air-Cool Planet, adds that the VCS and other standard-setting organizations are “doing good, hard work to move this system forward in a sustainable way.” He adds, “Offsets are a new concept, and they’re not well understood. What we’re seeing is progress toward a healthy market that for the moment has to police itself, but which is probably headed toward more regulation as the United States enters a compliance system for greenhouse gas reductions. If we don’t have an offset market, it’s really going to hamper our long-term ability to deal with climate change.”

Nevertheless, at the same time that regulators and certification groups are addressing questions of fraud, the offset market can be seen as holding the climate hostage, demanding payment before emissions are reduced. Steve McDougal, executive vice president of marketing and business development at offset provider 3Degrees, acknowledges this is a “strange dynamic.” But he suggests the market can sort out these issues.

“It’s a difficult thing, no doubt,” McDougal says. “But we’re encouraged when we see carbon financing make a project feasible. The idea here is that we can use carbon financing to make sustainable development cost-effective—that way we can make real progress on greenhouse gas reductions.” Indeed, despite gloomy assessments from Stanford University and the GAO, not to mention a barrage of negative press reports warning of fraud, some market experts believe offsets can play an important role in fighting climate change.

## Explaining the Nuts and Bolts

Offsets have their roots in the Kyoto Protocol, which came into force 16 February 2005. As part of the protocol, signatories agree to cap greenhouse gas emissions generated by participating countries. Industry sectors within those economies are given or sold emissions “allowances,” which they can trade to reach specified reduction targets. This “cap-and-trade” system—now the world’s dominant market approach for fighting anthropogenic climate change—is also embraced by RGGI in the United States. Moreover, the Western Climate Initiative, another North American regional coalition, plans to establish a cap-and-trade program to meet its climate goals. The Western Climate Initiative encompasses the states of Arizona, California, New Mexico, Oregon, Utah, and Washington and the Canadian provinces of British Columbia and Manitoba.

Allowances under any cap-and-trade program are distributed by their constituent regulated economies. For instance, under the Kyoto Protocol, allowances are either sold, given away, or auctioned by the countries that signed the treaty, or in the case of the European Union, by the Greenhouse Gas Emission Trading Scheme.

To understand how offsets and allowances differ, one first needs to understand how cap-and-trade works. Here’s a simplified explanation: Assume the combined annual emissions from a hypothetical participating economy comprising 10 greenhouse gas emitters is capped at 10 tons per year. Under a cap-and-trade system, the government of that economy might issue each emitter an annual emissions allowance of 1 ton. Now, let’s say one of those emitters, Company X, knows it will generate 2 tons of carbon dioxide. To reach its mandated 1-ton target, Company X can either reduce emissions (for example, by making operations more efficient or using cleaner fuels), or it can buy an allowance from Company Y, whose emissions, in this hypothetical world, are zero. If that allowance costs less than the price of new plant modifications, Company X has a money-saving opportunity—it can buy Company Y’s allowance and keep what it otherwise would have spent on plant upgrades. Company Y, meanwhile, has the opportunity to profit from its investment in clean technology.

In the event that allowances under a cap-and-trade system become too expensive—perhaps because officials tighten the emissions cap—emitters bound to mandated reductions can buy cheaper offsets from projects elsewhere in the world. But given that cap-and-trade systems aim to promote cleaner technology at home, and because allowances can be measured and verified more easily than offset projects, cap-and-trade programs typically limit the number of offsets companies can buy. RGGI, for instance, limits offset purchases to 10% or less of compliance obligations.

## Questions over Forestry

Of all the types of offset schemes available, those involving forest resources, particularly in tropical countries, have raised some of emissions the most challenging issues. CO_2_ from tropical deforestation and wood burning account for 17.3% of greenhouse gas releases to the atmosphere, according to a report in the 18 May 2007 issue of *Science*. That’s slightly more than the global road transportation sector, as calculated in the 8 December 2008 issue of *JAMA*. For that reason, efforts to fight global warming must consider forestry, says Janet Peace, vice president of markets and business strategy at the Pew Center on Global Change. But regulators disagree as to which forestry practices meet the criteria for viable offset projects.

The CDM allows for two types of forestry offsets: reforestation of previously harvested areas and afforestation, or tree planting where forests haven’t existed for at least 50 years. A third type of offset, avoided deforestation, deals with efforts to stop tree harvesting or clearing that would otherwise occur. However, the CDM does not include avoided deforestation activities. One reason for this is that when the Kyoto Protocol was first drafted, methodologies and cost-effective technologies for establishing baseline tropical forest cover and for monitoring forestry practices over time were not sufficiently developed, explains Toby Janson-Smith, senior director for forest carbon markets at Conservation International, an environmental group based in Arlington, Virginia. Without solid baseline and project-monitoring data and methodologies, policy makers weren’t confident in their ability to confirm that swaths of forest supposedly protected by offsets would have otherwise been cut down or to account for the number of carbon credits being claimed.

Today, thanks to advances in remote sensing and satellite imaging, and the emergence of new carbon accounting methodologies, those baselines and project practices can now be accurately documented and monitored, Janson-Smith says. Given that, he adds, policy makers are now working out how they might include avoided deforestation as an acceptable offset activity in the second Kyoto Protocol commitment period that begins in 2013.

But consensus on how forest offsets should be applied is scarce. RGGI, for instance, only credits local forest offsets from within states participating in the initiative. A Legislative Discussion Draft issued 7 October 2008 by John Dingell (D–MI) and Rick Boucher (D–VA) limits its proposed offsets to afforestation and reforestation only. And Green-e Climate doesn’t endorse any of the CDM’s forestry offsets. Jane Valentino, who manages the Green-e Climate program, says that’s because the CDM’s executive board views forestry offsets as temporary, “which makes sense for a compliance market, because the onus is on regulated entities to make sure that if a forest burns down, they’ll go buy another offset to replace it,” she explains. “But that doesn’t work for a voluntary market, because you can’t expect consumers to continue checking to make sure the forests are still there.”

Green-e Climate circumvents this particular problem by endorsing only forestry credits that have been certified by the VCS. Under the VCS system, forest projects are assessed to determine the likelihood that their stored carbon could be lost in the future. Offset credits from higher-risk projects (those with greater potential for future deforestation) are split into two portions. One portion is sold, while the rest is deposited into a pooled buffer account administered by the VCS. That way, the amount of carbon sequestered within a higher-risk forest exceeds the tons of carbon offsets sold for its protection to the buyer. As sales of forest offsets grow, so does the size of the risk pool, which covers the carbon lost if a protected forest is burned or harvested.

Janson-Smith says forestry offsets have value beyond slowing climate change. “More than 80% of the world’s poor depend on forest resources for their survival,” he says. “With every acre of forest lost, these local communities are further marginalized. And tropical forests contain most of the world’s threatened plants and animals. So through forest offsets, we can sustain the poor, prevent species loss, and slow climate change all at the same time. We’re losing fifty thousand square miles of tropical forest every year. Carbon financing is one of the only ways we can turn the tide on deforestation; there simply isn’t funding to do it otherwise.”

## Offsets in U.S. Climate Change Policy

It’s still unclear how offsets could factor into a national cap-and-trade system for the United States. S. 2191, the Climate Security Act of 2007, spearheaded by Joseph Lieberman (I–CT) and John Warner (R–VA), would have limited offset purchases under its proposed cap-and-trade plan to 30%, half from domestic projects and half from projects overseas. The bill failed to pass the Senate over persistent questions about cost containment, according to Peace. The Dingell–Boucher Discussion Draft issued in October limits offsets to just 5% of compliance obligations, split between domestic and international projects. But Dingell and Boucher’s offset fraction also grows as the emissions cap becomes increasingly stringent.

A tightening cap would drive allowance costs higher, making the cheaper offsets increasingly useful, some legislators believe. Given questions over offset reliability and the potential that investing in far-flung projects would deny local populations the health benefits of cleaner technology, fights over offsets in climate change policy are already erupting on Capitol Hill, says Emily Figdor, the federal global warming program director for the advocacy group Environment America.

At the same time, James Hansen, former head of the National Aeronautics and Space Administration’s Goddard Institute for Space Studies, and a leading advocate for climate change legislation, has rejected emissions trading altogether in favor of a carbon tax. In an open letter delivered on 29 December 2008 to then President-Elect Obama and his wife, Hansen and wife Anniek described cap-and-trade schemes as “ineffectual and not commensurate with the climate threat.” What’s needed instead, the Hansens insisted, are moratoriums on coal plants that don’t capture and sequester CO_2_, a carbon tax set by governments that rises over time, and “fourth-generation” nuclear power plants that burn their own waste.

The Hansens are not alone in rejecting offsets. In the 24 January 2009 issue of *New Scientist*, environmentalist James Lovelock, who proposed the “Gaia hypothesis” that the Earth constitutes a single superorganism, said, “Carbon trading, with its huge government subsidies, is just what finance and industry wanted. It’s not going to do a damn thing about climate change, but it’ll make a lot of money for a lot of people and postpone the moment of reckoning.” As for what he does see as an answer to climate change, Lovelock said, “There is one way we could save ourselves and that is through the massive burial of charcoal. It would mean farmers turning all their agricultural waste—which contains carbon that the plants have spent the summer sequestering—into nonbiodegradable charcoal and burying it in the soil. Then you can start shifting really hefty quantities of carbon out of the system and pull the CO_2_ down quite fast.” [For more on this technology, see “Biochar: Carbon Mitigation from the Ground Up,” p. A70 this issue.]

The imperative, stresses Lewis Milford, president of the Clean Energy Group, a Vermont firm that seeks to accelerate the renewable energy market, is that any focus on offsets not slow the development of breakthrough technologies needed to reverse climate change quickly. Milford says carbon pricing—either through cap-and-trade or a direct tax on carbon—may not do enough to spur new technology.

“We’re seeing a consensus that cap-and-trade won’t generate the kind of high prices on carbon that we need [to drive policy change], especially given political challenges and the current state of the economy,” Milford says. “If you’re looking at this from the perspective of needing breakthrough, expensive systems—for instance carbon capture and storage, or advances in solar energy—I don’t think incremental price incentives are going to get you there. We need technology programs and significant public investments. Cap-and-trade could have some value, but it’s likely to be limited if carbon prices stay low and volatile.”

## Figures and Tables

**Figure:**
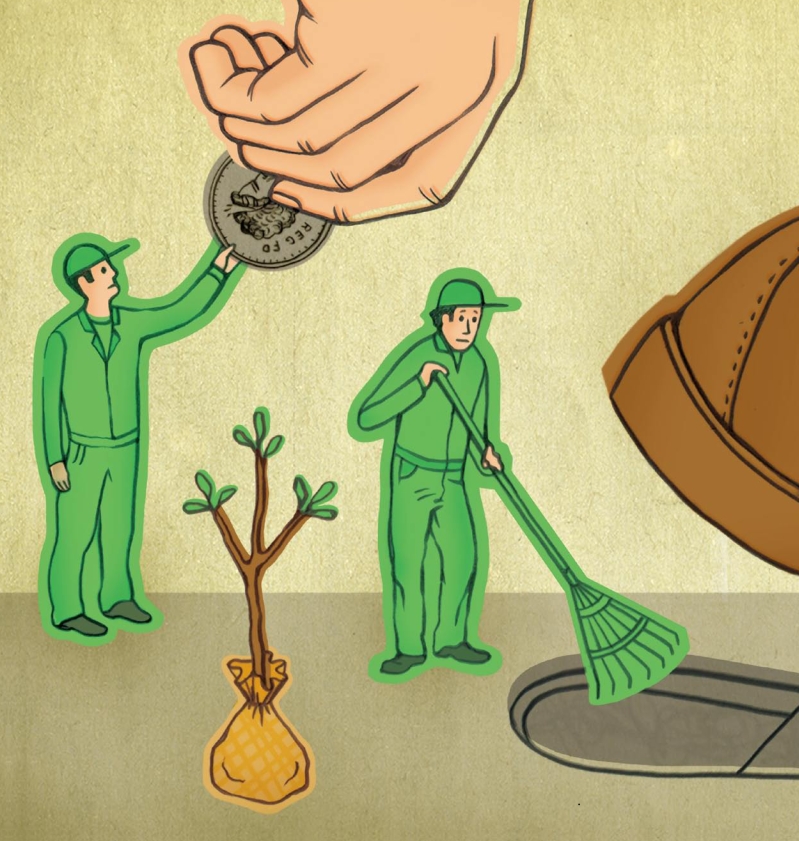


**Figure:**
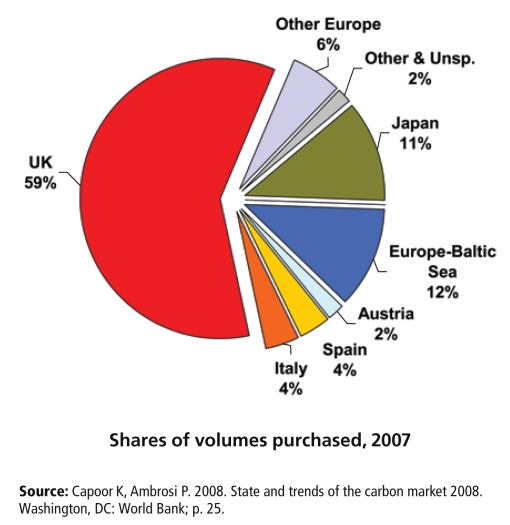
Countries Purchasing Offsets to Meet Mandated Emissions Reductions

**Figure:**
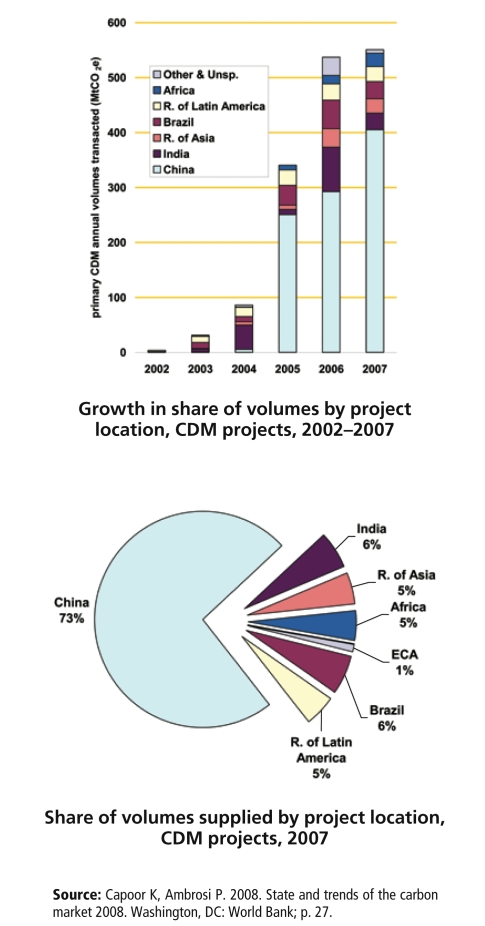
Where Are Offset Projects Taking Place?

**Figure:**
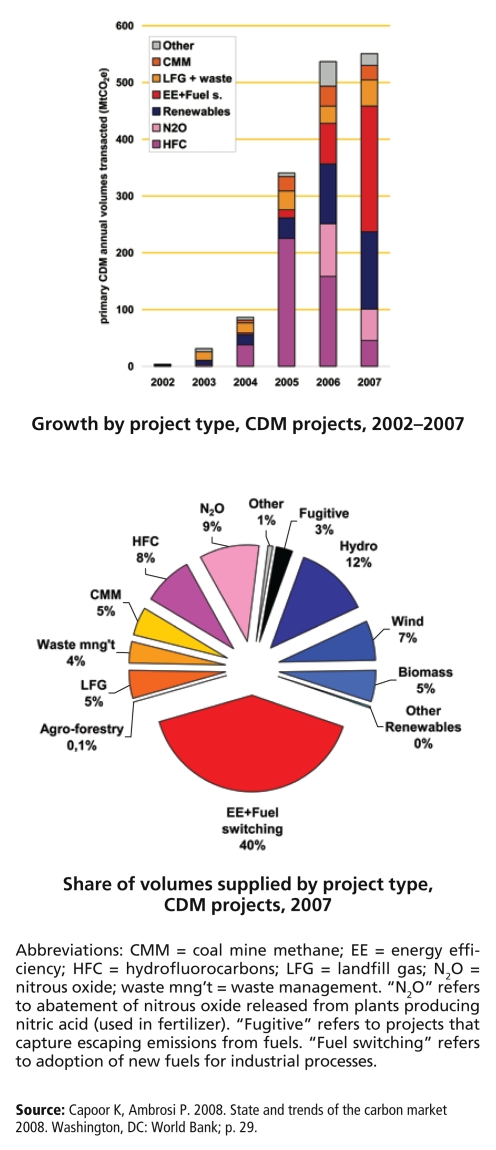
Types of Offset Projects

**Table:** Transaction volume by project location, over-the-counter projects, 2007

Asia	39%
North America	27%
Europe and Russia	13%
Australia	7%
Latin America	7%
Other	5%
Africa	2%

**Adapted from:** Hamilton K, Sjardin M, Marcello T, Xu G. 2008. Forging a frontier: state of the voluntary carbon markets 2008. San Francisco, CA: Ecosystem Marketplace; p. 7.

**Table:** Transaction volume by project type, over-the-counter projects, 2007

Renewable energy	31%
Energy efficiency	18%
Forestry	18%
Methane destruction	16%
Fuel switching	9%
Mixed	5%
Industrial gas destruction	2%
Geological sequestration	1%

**Adapted from:** Hamilton K, Sjardin M, Marcello T, Xu G. 2008. Forging a frontier: state of the voluntary carbon markets 2008. San Francisco, CA: Ecosystem Marketplace; p. 7.

